# Mutational signature analysis in non-small cell lung cancer patients with a high tumor mutational burden

**DOI:** 10.1186/s12931-021-01871-0

**Published:** 2021-11-24

**Authors:** Guus R. M. van den Heuvel, Leonie I. Kroeze, Marjolijn J. L. Ligtenberg, Katrien Grünberg, Erik A. M. Jansen, Daniel von Rhein, Richarda M. de Voer, Michel M. van den Heuvel

**Affiliations:** 1 Department of Pulmonology, Radboud University Medical Center, Postbox 9101, 6500 HB Nijmegen, The Netherlands; 2 Department of Pathology, Radboud University Medical Center, Nijmegen, The Netherlands; 3 Department of Human Genetics, Radboud University Medical Center, Nijmegen, The Netherlands

**Keywords:** Mutational signatures, Next generation sequencing, Non-small cell lung cancer, Cancer genetics

## Abstract

**Background:**

Lung cancer is the leading cause of cancer death worldwide. With the growing number of targeted therapies and the introduction of immuno-oncology (IO), personalized medicine has become standard of care in patients with metastatic disease. The development of predictive and prognostic biomarkers is of great importance. Mutational signatures harbor potential clinical value as predictors of therapy response in cancer. Here we set out to investigate particular mutational processes by assessing mutational signatures and associations with clinical features, tumor mutational burden (TMB) and targetable mutations.

**Methods:**

In this retrospective study, we studied tumor DNA from patients with non-small cell lung cancer (NSCLC) irrespective of stage. The samples were sequenced using a 2 megabase (Mb) gene panel. On each sample TMB was determined and defined as the total number of single nucleotide mutations per Mb (mut/Mb) including non-synonymous mutations. Mutational signature profiling was performed on tumor samples in which at least 30 somatic single base substitutions (SBS) were detected.

**Results:**

In total 195 samples were sequenced. Median total TMB was 10.3 mut/Mb (range 0–109.3). Mutational signatures were evaluated in 76 tumor samples (39%; median TMB 15.2 mut/Mb). SBS signature 4 (SBS4), associated with tobacco smoking, was prominently present in 25 of 76 samples (33%). SBS2 and/or SBS13, both associated with activity of the AID/APOBEC family of cytidine deaminases, were observed in 11 of 76 samples (14%). SBS4 was significantly more present in early stages (I and II) versus advanced stages (III and IV; *P* = .005).

**Conclusion:**

In a large proportion of NSCLC patients tissue panel sequencing with a 2 Mb panel can be used to determine the mutational signatures. In general, mutational signature SBS4 was more often found in early versus advanced stages of NSCLC. Further studies are needed to determine the clinical utility of mutational signature analyses.

**Supplementary Information:**

The online version contains supplementary material available at 10.1186/s12931-021-01871-0.

## Background

Lung cancer is the leading cause of cancer-related death in many countries[1]. Its high mortality has urged major efforts to optimize the treatment of lung cancer have been made that have resulted in targeted therapies and immunotherapy. These treatments are based on the presence or absence of specific predictive and prognostic biomarkers. In the last decade several biomarkers predicting immune checkpoint blockade outcomes have been discovered. One of these biomarkers is the expression of PD-L1 on tumor cells, indicating eligibility for immune checkpoint inhibitor therapy [2, 3]. However, PD-L1 expression as a predictive biomarker for response to immune checkpoint blockade (ICB) is fairly unreliable due to dynamic and heterogeneous expression in the tumor microenvironment, divergent assay interpretation and lack of PD-L1 platform standardization [4–8]

The first FDA-approved tumor type-agnostic biomarker for immunotherapy is microsatellite instability (MSI) [9]. MSI is caused by inactivation of the mismatch repair (MMR) machinery, resulting in the accumulation of DNA replication errors in repetitive sequences or microsatellites. Alternatively, TMB is suggested as a biomarker, as the number of mutations observed in a tumor seem to correlate with clinical outcome and effectiveness of immunotherapy [10–12]. However, TMB is not yet approved as a predictive biomarker for NSCLC as recent studies demonstrated varying results regarding therapeutic benefits [13, 14].

A relative new strategy in the search for new biomarkers is to study the molecular processes in the cancer cell that cause a specific pattern of mutations; a so-called *mutational signature*. Somatic mutations in a cancer genome are the cumulative result of mutational processes that started since embryonic development [15]. Different mutational processes, such as exposure to UV-light or tobacco smoking, generate a unique combination of mutation types, that can be detected as mutational signatures. Mutational signatures have shown their applicability in cancer diagnosis and prediction of response to treatment [16–18]. Here we investigated mutational signature analyses in a retrospective cohort of NSCLC patients who underwent comprehensive genomic tumor profiling.

## Methods

### Sample and data collection

**Fig. 1:**
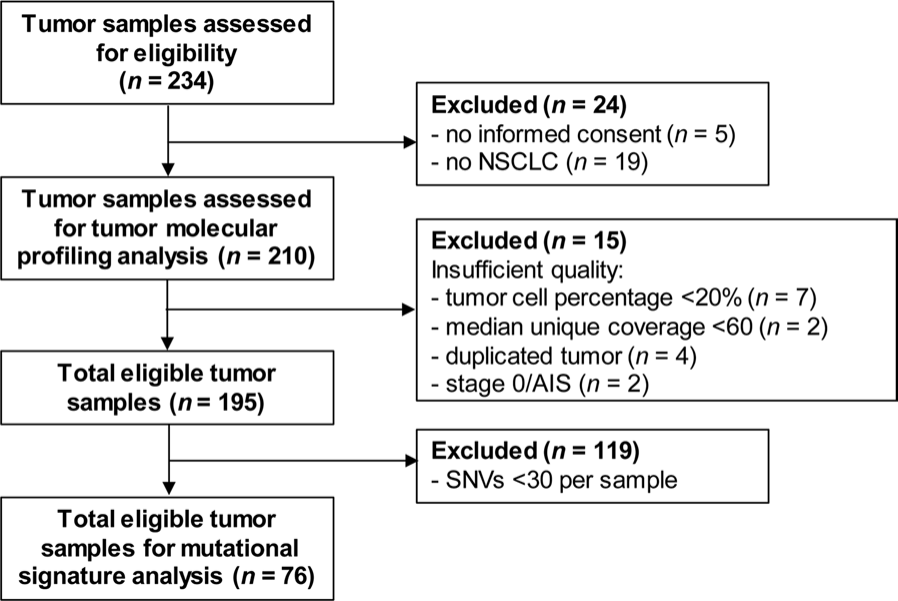
Flowchart of the study. *NSCLC* non-small cell lung cancer, *AIS* adenocarcinoma in situ, *SNV* single nucleotide variants. * In one sample molecular sequencing analysis was already performed in the context of the TSO500 validation trial written by Leonie Kroeze et al. [23]

We included 210 samples from patients diagnosed with NSCLC at the Radboud university medical center (Nijmegen, the Netherlands). Samples were sequenced between March 2019 and March 2020. Seven tumor samples were taken before 2019. We included tumor samples with a final diagnosis of NSCLC of any stage either derived directly from the lungs (lobectomy, pneumonectomy, lung or bronchial biopsy and bronchoalveolar lavage) or derived from a metastatic locus (Additional file 1: Table S1). Only one sample per solitary tumor was included. We excluded samples with low tumor cell percentages (< 20%), low median unique coverage (< 60), duplicate tumors and stage 0 disease (carcinoma in situ). A total of 195 tumor samples from 192 patients were used for further analysis. The study eligibility criteria included patients with histological or cytological confirmation of NSCLC (Fig. 1).

The study was conducted in accordance with the institutional guidelines and regulations from the Radboud university medical center. Written informed consent was obtained for all patients. We obtained the electronic medical records of all these patients and extracted age, gender, smoking status, stage, Eastern Cooperative Oncology Group (ECOG) performance score at baseline and treatment modality.

### Tumor sequencing and analyses

All tumor samples were subjected to sequencing analysis using TSO500 (Illumina), a next-generation sequencing panel containing 523 cancer related genes (total size: 2 Mb), performed either as part of standard care in patients with advanced NSCLC or for specific study purposes (for example the LEMA trial) [19]. Sequencing libraries were prepared using the hybrid capture-based TSO500 library preparation kit following the manufacturer’s protocol. After quantification, normalization and pooling, the libraries were sequenced on a NextSeq 500 (Illumina), with 10 libraries sequenced per run (NextSeq high output). The sequence data were processed and analyzed by the TruSight Oncology 500 Local App version 1.3 or 2.0 (Illumina). Analyses of single nucleotide variants (SNVs), multiple nucleotide variants (MNVs), copy number variants (CNVs), microsatellite instability (MSI) and tumor mutational burden (TMB) were performed. A tumor was considered MSI positive when at least 25% of accessible MSI sites are unstable. TMB was defined as the number of synonymous and nonsynonymous mutations with a variant allele frequency of at least 5% per Mb of sequence. We used 10 mut/Mb as cut-off value based on large-base clinical studies [20, 21]. Patient characteristics were investigated according to TMB status (Additional file 2: Table S2). For tailored reporting purposes, a gene panel of 15 genes relevant for lung cancer was investigated for relevant actionable mutations (Additional file 2: Table S3). Tumor samples with at least 30 somatic single base substitutions were investigated by means of mutational signature analysis using COSMIC mutational signatures v3 (n = 76) as described by Kroeze et al. [22]^23^.

### Statistics

Differences between groups were calculated using the Fisher's exact test. A p-value of < 0.05 is considered significant.

## Results

### Clinical and histopathologic features of NSCLC

In total, 195 NSCLC samples eligible for inclusion in this study were molecularly profiled (Fig. 1). Mean age of diagnosis was 67 years (range 34–88) and 54.8% of the patients were male. Most tumors were stage IV (*n* = 98; 50.3%), followed by stage III (*n* = 44; 22.5%), stage I *(n* = 30; 15.4%) and stage II (*n* = 23; 11.8%). Review of histopathology showed that the majority of tumors represented adenocarcinoma (AC, *n* = 110; 56.4%), followed by squamous cell carcinoma (SCC, *n* = 42; 21.5%) and large cell neuroendocrine carcinoma (LCNEC, *n* = 11; 5.6%). A history of smoking, active or former, was reported for 98.9% of cases (*n* = 193). For 84% of the tumors PD-L1 status was investigated (*n* = 164). PD-L1 expression was negative in 80 tumors (48.8%), intermediate (1–50% positive stained tumor cells) in 32 tumors (19.5%) and high (above 50% positive stained tumor cells) in 52 tumors (31.7%) (Additional file 2: Table S2).

### High tumor mutational burden is associated with lower tumor stages and specific driver gene mutations

The median unique exon coverage was 474 (range 64–851). MSI could be assessed in 168 tumors (86.2%), but none showed MSI (Additional file 1: Table S1). The median overall total TMB was 10.3 (range 0.0–109.3; Additional file 2: Figure S2A). Median total TMB varied among different disease stages. Stage IV tumors presented with a lower TMB (median TMB 9.5) than tumors in earlier stages of disease (median TMB 10.2; *P* = 0.013, 13.9; *P* = 0.003, and 13.4; *P* = 0.051, for stage I, II and III, respectively; Additional file 2: Table S2; Additional file 2: Figure S2B). Moreover, median total TMB was higher in tumors with PD-L1 expression (> 1% positive tumor cells) than tumors that did not express PD-L1 (median TMB 13.0 vs 9.5, respectively; *P* = 0.004), of which tumors with high PD-L1 expression (> 50 positive tumor cells) had the highest TMB (median TMB 14.2; *P* = 0.001; Additional file 2: Figure S2C). Age appeared to be lower in the high TMB group (> 10 mut/Mb) (*P* = 0.025). No correlations between TMB and sex or histopathological subtypes were observed (Additional file 2: Table S2).

Furthermore, we investigated somatic driver mutations with clinical relevance for NSCLC patients (Additional file 2: Table S3). *TP53* mutations were more frequent in the TMB high group versus TMB low group (*P* = 0.007). We also noted a higher frequency of *STK11* mutations in the TMB high group (*P* = 0.012).

### Mutational signature SBS4 is associated with lower tumor stages

**Fig. 2:**
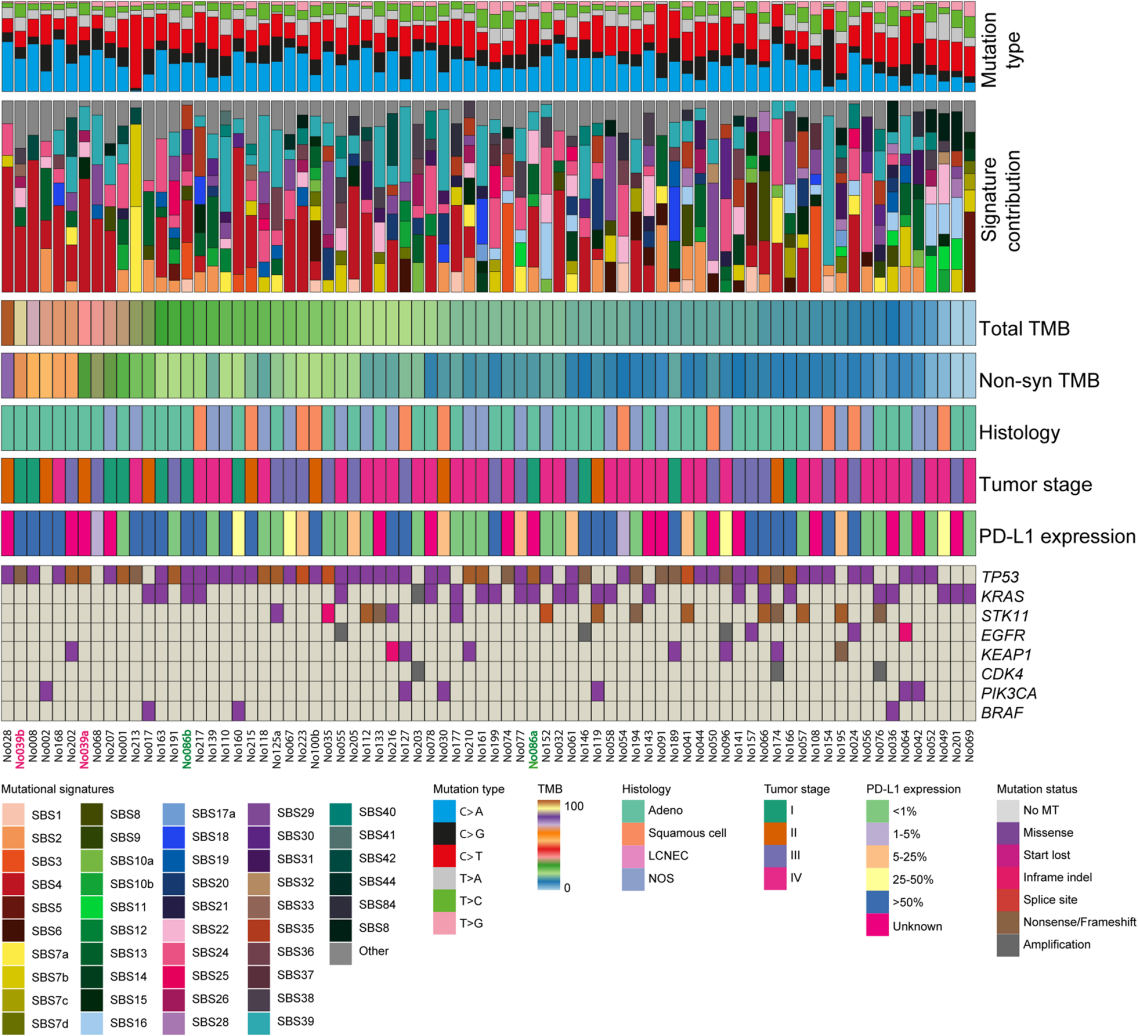
Clinicopathological and molecular features of lung tumor samples with ≥ 30 somatic SBS in genes covered by the TSO500 panel. Samples are sorted from left-to-right based on high-to-low total TMB. Every column represents one tumor sample. Numbers indicated in purple and green indicate two independent tumors (not clonally related) from the same individual. Only lung cancer related genes in which 2 or more somatic mutations or amplifications were detected are included. *TMB* tumor mutational burden, *Non-syn* non-synonymous, *MT* mutation, *NOS* not otherwise specified, *LCNEC* Large cell neuroendocrine carcinoma, *SBS* single base substitution

We analyzed the mutation types and mutational signatures of tumor samples with at least 30 SBS (n = 76; Table 1). The median TMB of this set of tumors was 15.2 mut/Mb. The mutation types observed in the tumors were mostly C > A and C > T mutations (Fig. 2). By refitting of mutational signatures (COSMIC v3) we could assign the majority of somatic mutations detected in the tumor samples to a known mutational signature (average cosine 0.77; range 0.50–0.96; Additional file 1: Table S1). SBS signature 4 (SBS4), associated with tobacco smoking, contributed to the mutation spectrum in 25 of 76 tumors (33%) with a relative contribution of at least 20%. All patients showing SBS4 had a smoking history, among which one patient only reported passive smoking during decennia. SBS signatures 2 and/or 13 (SBS2/13), both associated with activity of the AID/APOBEC family of cytidine deaminases, cumulatively contributed to the mutation spectrum in 11 of 76 tumors (14%). SBS signature 29 (SBS29), associated with tobacco chewing, contributed to the mutation spectrum in 5 of 76 tumors (7%). The contribution of each of these mutational signatures was almost mutually exclusive, with the exception of one sample in which both SBS4 and SBS2/13 were detected with a relative contribution of more than 20% (Additional file 1: Table S1; Fig. 2). Furthermore, SBS signature 39 (SBS39), a signature with unknown etiology, likely contributed to the mutation spectrum in 11 of 76 tumors (14%). In one tumor (T213, TMB 26 mut/Mb) the mutation spectrum was almost solely explained by signatures associated with exposure to UV light (SBS7a and SBS7b; Fig. 2). Clinical and pathological revision revealed that this patient presented with an ulcerative skin lesion with malignant properties, issued as a lung metastatic lesion. Histopathological analysis performed on endobronchial biopsies pointed in the direction of a keratin positive NSCLC not otherwise specified. However, we found pathological mutations in the *PTPN11, NF1, CUX1, IKZF1* gene and *TERT* promoter, which better fit a diagnosis of a melanoma than of lung cancer.

**Table 1:** Patient characteristics, according to mutational signature status

Characteristic	All patients (*n* = 76)	SBS4 (*n* = 25)	SBS2/13 (*n* = 11)	SBS29 (*n* = 5)	Other (*n* = 37)
Age at enrollment—years					
Mean ± SD	63.7 ± 9.8	63.7 ± 9.2	67.9 ± 8.4	69.8 ± 9.6	65.1 ± 10.6
Median (range)	67 (34–87)	62 (45–80)	68 (52–81)	75 (58–79)	68 (34–87)
Sex—no. (%)					
Female	41 (54)	14 (56)	6 (55)	3 (60)	14 (38)
Male	35 (46)	11 (44)	5 (45)	2 (40)	23 (62)
Histological diagnosis—no. (%)					
Adenocarcinoma	43 (57)	17 (68)	7 (64)	3 (60)	18 (49)
Squamous-cell carcinoma	11 (14)	3 (12)	1 (9)	1 (20)	6 (16)
Large cell neuroendrocrine carcinoma	4 (5)	1 (4)	0 (0)	0 (0)	3 (8)
Other	18 (24)	4 (16)	3 (27)	1 (20)	10 (27)
Clinical stage—no. (%)					
I	11 (14)	8 (32)	3 (27)	1 (20)	0 (0)
II	9 (12)	4 (16)	2 (18)	0 (0)	3 (8)
III	20 (27)	5 (20)	1 (9)	3 (60)	12 (32)
IV	36 (47)	8 (32)	5 (46)	1 (20)	22 (60)
Smoking status—no. (%)					
Active or former	72 (95)	24 (96)	11 (100)	5 (100)	34 (92)
Never	3 (4)	1 (4)	0 (0)	0 (0)	2 (5)
Unknown	1 (1)	0 (4)	0 (0)	0 (0)	1 (3)
PD-L1 status – no. (%)					
< 1%	22 (29)	5 (20)	4 (36)	1 (20)	12 (32)
1–50%	13 (17)	5 (20)	0 (0)	1 (20)	8 (22)
> 50%	25 (33)	10 (40)	4 (36)	2 (40)	9 (24)
Unknown	16 (21)	5 (20)	3 (28)	1 (20)	8 (22)

Next, we investigated specific mutational signatures in relation to tumor characteristics and driver gene mutations. SBS4 was more present in early stages (I and II) versus advanced stages (III and IV; *P* = 0.005). Samples harboring SBS4 had a higher median TMB than samples with SBS2/13, SBS29 or other contributions (Additional file 2: Figure S1). PD-L1 status did not differ among SBS4, SBS2/13 and SBS29 (Additional file 2: Table S3). *TP53* mutations were more often seen in tumors with a high relative contribution of signature SBS4 compared to tumors in which the contribution of SBS4 was low or absent (*P* = 0.002) (Additional file 2: Table S4). Other driver mutations were not associated with the presence of SBS4, SBS2/13 or SBS29 in a tumor. No difference was noticed regarding samples with SBS4 versus all other samples regarding sampling locus or histological subtype (AC or SCC).

## Discussion

Here, we investigated tumor mutational burden and mutational signatures in a clinical cohort of NSCLC. Using a 2 Mb gene panel in 39% of tumors sufficient single base substitutions were detected to describe the mutational signatures. SBS4 and SBS2/13 were detected in 33% and 14%. SBS4 appears to be more common in early stages of NSCLC. In addition, mutational signature analyses led to the re-classification of a NSCLC as a metastasis of a tumor that likely originated from the skin.

By mutational signature refitting in samples with a high TMB we observed a major contribution of SBS4, SBS2/13 and SBS29 as one would expect in NSCLC [24]. A major proportion of somatic mutations is known to be formed by exogenous exposures like tobacco smoking in lung cancer. Furthermore, genomic alterations introduced by smoking persist for many years after smoking cessation [25]. Previously, Rizvi and colleagues found a link between signature SBS4 and progression-free survival in patients with NSCLC who received Pembrolizumab. These data have not yet been confirmed in other studies but reveal the potential of qualitative analysis of mutation patterns in relation to treatment outcome. The apolipoprotein B mRNA editing enzyme, catalytic polypeptide-like (APOBEC) related signatures SBS2 and SBS13 are frequently observed in our population. The presence of these mutational signatures is markedly associated with response to immune checkpoint blockade (ICB) therapy [26]. It is suggested that the cellular machinery underlying SBS2 and SBS13 is activated by tobacco smoke via direct or indirect pathways [27]. The clinical implications of our study need further assessment in larger series of clinical lung cancer samples in which TMB and mutational signature analyses can be combined with long-term follow-up data on treatment and survival.

In one tumor sample SBS7a and SBS7b could fully explain the mutation spectrum, which suggests that this tumor originates from sun exposed skin [24]. SBS7 mutational signatures are normally seen in skin cancers such as melanoma [28]. Analyzing mutational signatures in NSCLC can as such contribute to assess the primary tumor site of the malignancy.

This is the first study that describes higher TMB levels in early NSCLC stages versus stage IV. In a systematic review of Willes regarding TMB and lung cancer, four publications assessed lung cancer stage, all reporting no significant association with TMB [29]. Within this review two publications referred to patients with SCC or LCNEC/SCLC only [30, 31], whereas the remaining two articles referred to patients with AC [32, 33]. The clonal structure of a tumor varies considerably between primary and metastatic sites, with higher rates of monoclonal structures recorded in metastases due to clonal selection [34]. As such, TMB can be measured from a primary or metastatic tumor sample, causing systematic bias in TMB values.

To our knowledge, this is the first study showing signature SBS4 is more frequently detected in early versus advanced stages of NSCLC. An explanation for this finding could be the higher risk of cardiovascular and pulmonary disease due to tobacco smoking, which often leads to relatively early imaging diagnostics.

Since Alexandrov and Stratton uncovered and catalogued mutational signatures, our understanding of the mutational processes that cause somatic mutations is markedly expanded [15]. However, the understanding of mutational processes in most cancer types is remarkably limited. Refitting of mutational signatures in clinical samples may provide additional insights into the processes underlying cancer development. However, as we have seen in our study this is only feasible when sufficient somatic mutations are detected in a tumor sample. We have applied the criterium of 30 SBSs and a minimal relative contribution of 20% to conclude that a mutational mechanism has been active in a tumor. Using a next gen sequencing panel covering 2 Mb in our population 39% of tumor samples could be evaluated for mutational signature analysis. When whole exome sequencing (WES) or whole genome sequencing (WGS) can be applied, more mutations are detected per tumor resulting in a higher percentage of samples suitable for mutational signature analysis. However, WES or WGS are not yet part of standard of care in cancer diagnostics. Additional studies are needed to assess the clinical utility of mutational signature analyses and to set guidelines to further translate findings of tumor-specific mutational processes into clinical practice.

In conclusion, this is the first report that systematically studies mutational signatures in a set of tumors sequenced in routine clinical practice in NSCLC. With a panel covering about 500 genes mutational signatures can be determined in a significant proportion of NSCLC. Interestingly, mutational signature SBS4 was more common in early versus advanced stages of NSCLC. Furthermore, mutational signature profiling may facilitate the diagnosis of the primary tumor site in a clinical setting. However, studies of greater magnitude and/or with other sequencing strategies using WES/WGS are needed to assess the clinical utility and to translate our findings into clinical practice.

## Supplementary Information

**Additional file 1: Table S1.** Clinical, histopathological and molecular features of non-small cell lung cancer samples.

**Additional file 2.** Additional figures and tables.

## Data Availability

All patients are known in the outpatient clinic of Pulmonology at the Radboud university medical center. Most of the included patients have provided informed consent for Lung cancer Early Molecular Assessment trial (LEMA) (clinicaltrial.gov: NCT02894853) or Lung cancer biobank (NCT01084785) or gave otherwise permission for using body specimens for research purposes. Missing informed consents for living patients have been collected.

## Abbreviations

AC: Adenocarcinoma
AID/APOBEC: Activation-induced deaminase/apolipoprotein B mRNA-editing enzyme, catalytic polypeptide
CNVs: Copy number variants
COSMIC: Catalogue of somatic mutations in cancer
ECOG: Eastern cooperative oncology group
ECRF: Electronic case report form
FDA: Food and drug administration
ICB: Immune checkpoint blockade
IO: Immuno-oncology
LCNEC: Large cell neuroendocrine carcinoma
Mb: Megabase
MNVs: Multiple nucleotide variants
MSI: Microsatellite instability
Mut/Mb: Mutations per megabase
NSCLC: Non-small cell lung cancer
PD-L1: Programmed cell death- ligand 1
SBS: Single base substitute
SCC: Squamous cell carcinoma
SCLC: Small cell lung cancer
SNVs: Single nucleotide variants
TMB: Tumor mutational burden
WES: Whole exome sequencing
WGS: Whole genome sequencing
