# Supplementary material for: Mutational signature analysis in non-small cell lung cancer patients with a high tumor mutational burden
Source: Respir Res. 2021 Nov 24;22:302. doi: 10.1186/s12931-021-01871-0 (PMC8611965; doi:10.1186/s12931-021-01871-0)
Supplement: Supplementary file 2 — Additional file 2. Additional figures and tables. [file 12931_2021_1871_MOESM2_ESM.docx]

**Additional data to:**

**Mutational signature analysis in non-small cell lung cancer patients with a high tumor mutational burden**

Guus R.M. van den Heuvel, MDᵃ; Leonie I. Kroeze, PhDᵇ; Marjolijn J.L. Ligtenberg, PhDᵇ^,^ᶜ; Katrien Grünberg, PhDᵇ; Erik A.M. Jansen, BScᶜ; Daniel von Rhein, PhDᶜ; Richarda M. de Voer, PhDᶜ **; Michel M. van den Heuvel, MD, PhDᵃ **

*ᵃ Department of Pulmonology, Radboud university medical center, Nijmegen, Netherlands*

*ᵇ Department of Pathology, Radboud university medical center, Nijmegen, Netherlands*

*ᶜ Department of Human Genetics, Radboud university medical center, Nijmegen, Netherlands*

** These authors contributed equally to this work

Running title: Mutational Signatures in Lung Cancer Patients

Corresponding author: M.M. van den Heuvel, Department of Pulmonology, Radboud University Medical Center, Postbox 9101, 6500 HB, Nijmegen, The Netherlands. Email: michel.vandenheuvel@radboudumc.nl

Funding: The authors received no financial support for the research, authorship, or publication of this article

Conflict of interests: The participating authors have no conflicts of interest to declare


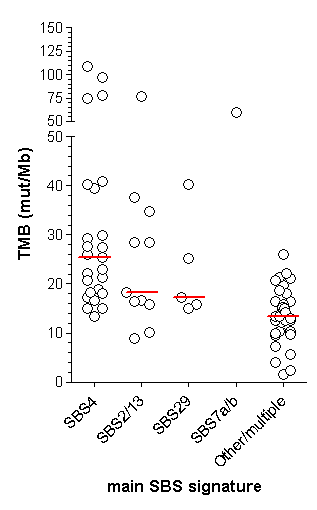


**Additional file 2: Figure S1: Tumor mutational burden (TMB) in samples for which mutational signatures could be determined.** When a single mutational signature contributed >20% in a tumor sample it was assigned to the specific SBS signature. Red line represents the median TMB.


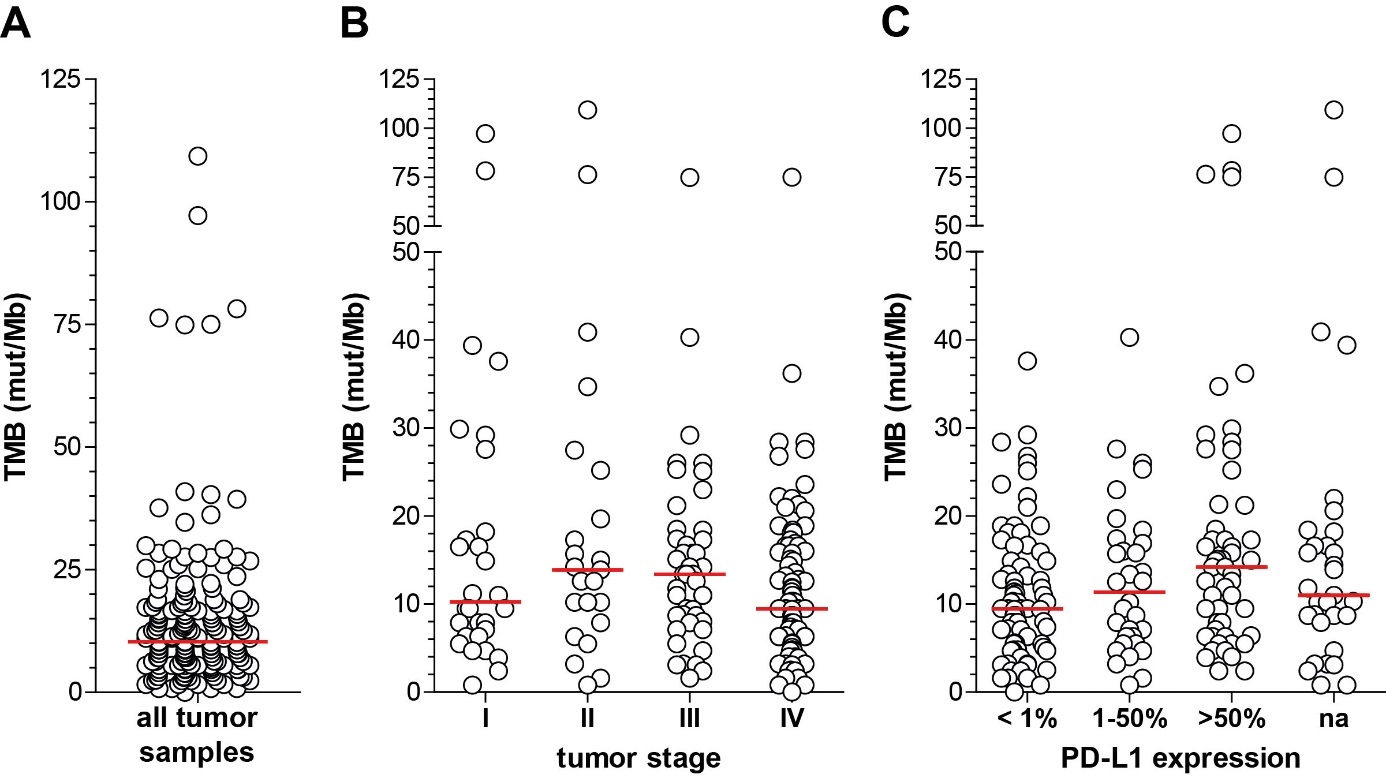


**Additional file 2: Figure S2: Tumor mutational burden (TMB) of non-small cell lung cancer samples.** **A)** TMB all tumor samples. **B)** TMB according to tumor stages. **C)**

TMB according to PD-L1 expression. Red line represents the median TMB.

**Additional file 2: Table S2: Patient and tumor characteristics according to TMB status**

| **Characteristic** | **All patients** | **Patients with total TMB < 10** | **Patients with total TMB ≥ 10** | ***P*-value**** |
| --- | --- | --- | --- | --- |
| Age at enrollment - years | | | | |
| Mean ± SD | 66.8 ± 9.2 | 68.4 ± 9.3 | 65.4 ± 9.0 | .0247 |
| Median (range) | 68 (34-88) | 70 (42-88) | 67 (34-84) |  |
| Sex - no. (%) | | | | |
| Female | 88 (45) | 40 (45) | 48 (55) | .8860 |
| Male | 107 (55) | 50 (47) | 57 (53) |  |
| Histologic diagnosis - no. (%) | | | | |
| Adenocarcinoma | 110 (56) | 57 (52) | 53 (48) | .08284 |
| Squamous-cell carcinoma | 42 (22) | 17 (40) | 25 (60) | .48540 |
| Large cell neuroendrocrine carcinoma | 11 (6) | 4 (36) | 7 (64) | .55090 |
| Other | 32 (16) | 12 (38) | 20 (62) | .33460 |
| Clinical stage - no. (%)* | | | | |
| I | 30 (15) | 15 (50) | 15 (50) | .69340 |
| II | 23 (12) | 6 (26) | 17 (74) | .04623 |
| III | 44 (23) | 16 (36) | 28 (64) | .16980 |
| IV | 98 (50) | 53 (54) | 45 (46) | .03128 |
| Smoking status - no. (%) | | | | |
| Active or former | 180 (92) | 81 (45) | 99 (55) | .29190 |
| Never | 13 (7) | 9 (69) | 4 (31) | .09370 |
| Unknown | 2 (1) | 0 (0) | 2 (100) | .50040 |
| PD-L1 status – no. (%) | | | | |
| <1% | 80 (41 | 44 (55) | 36 (45) | .04218 |
| 1-50% | 32 (16) | 15 (47) | 17 (53) | 1 |
| >50% | 52 (27) | 19 (37) | 33 (63) | .14340 |
| Unknown | 31 (16) | 12 (39) | 19 (61) | .43400 |

* According to the IASLC TNM classification 8^th^ edition

** Fisher’s exact test for number of samples in each category

TMB: tumor mutational burden, defined as number of somatic mutations per megabase

**Additional file 2: Table S3: Genes somatically mutated according to TMB status**

| **Gene** | **Total number of patients (%)** | **Median**  **TMB** | **Number of patients with total TMB < 10** | **Number of patients with total TMB ≥ 10** | ***P-*value*** |
| --- | --- | --- | --- | --- | --- |
| *TP53* | 138 (71) | 12.2 | 55 (28) | 83 (43) | 0.0073 |
| *KRAS* | 56 (29) | 11.4 | 25 (13) | 31 (16) | 0.8742 |
| *STK11* | 26 (13) | 14.2 | 6 (3) | 20 (10) | 0.0117 |
| *EGFR* | 20 (10) | 5.5 | 13 (7) | 7 (4) | 0.0975 |
| *KEAP1* | 15 (8) | 13.6 | 4 (2) | 11 (6) | 0.1767 |
| *PIK3CA* | 14 (7) | 9.5 | 7 (4) | 7 (4) | 0.7877 |
| *BRAF* | 7 (4) | 10.2 | 2 (1) | 5 (3) | 0.4545 |
| *FGFR1* | 4 (2) | 8.26 | 2 (1) | 2 (1) | 1 |
| *CDK4* | 3 (2) | 15.0 | 0 (0) | 3 (2) | 0.2506 |
| *ALK* | 3 (2) | 3.2 | 2 (1) | 1 (.5) | 0.5962 |
| *ERBB2* | 3 (2) | 11.2 | 1 (.5) | 2 (1) | 1 |
| *KIT* | 2 (1) | 11.0 | 1 (.5) | 1 (.5) | 1 |
| *PDGFRA* | 1 (.5) | 12.6 | 0 (0) | 1 (.5) | 1 |
| *HRAS* | 1 (.5) | 39.4 | 0 (0) | 1 (.5) | 1 |
| *CDK6* | 1 (.5) | 4.7 | 1 (.5) | 0 (0) | 0.4615 |
| *CCND1* | 1 (.5) | 17.3 | 0 (0) | 1 (.5) | 1 |
| *MET* | 1 (.5) | 17.3 | 0 (0) | 1 (.5) | 1 |

* Fisher’s exact test for number of samples in each category

TMB: tumor mutational burden, defined as number of somatic mutations per megabase

Amplification was established once in KRAS (13 copies), 4 times in EGFR (5, 8, 15 and 103 copies), 3 times in PIK3CA (10, 15 and 20 copies), twice in FGFR1 (7 and 10 copies), once in CDK4 (29 copies), once in ERBB2 (92 copies), once in KIT (16 copies), once in HRAS (10-20 copies) and twice in MET (12 and 15 copies).

**Additional file 2: Table S4: Genes somatically mutated according to presence of specific mutational signatures**

| **Gene (% mutated)** | **Total number of patients (*n* = 76)** | **SBS4**  **(*n* = 25)** | **SBS2/13**  **(*n* = 11)** | **SBS29**  **(*n* = 5)** |
| --- | --- | --- | --- | --- |
| *TP53* | 60 (79) | 22 (88) | 9 (82) | 3 (60) |
| *KRAS* | 25 (33) | 6 (24) | 3 (27) | 2 (40) |
| *STK11* | 15 (20) | 6 (24) | 0 (0) | 1 )20) |
| *EGFR* | 6 (8) | 2 (8) | 1 (9) | 0 (0) |
| *KEAP1* | 8 (11) | 2 (8) | 1 (9) | 0 (0) |
| *PIK3CA* | 6 (8) | 0 (0) | 3 (27) | 0 (0) |
| *BRAF* | 3 (4) | 1 (4) | 1 (9) | 0 (0) |
| *FGFR1* | 1 (1) | 0 (0) | 0 (0) | 1 (20) |
| *CDK4* | 3 (4) | 1 (4) | 0 (0) | 0 (0) |
| *ALK* | 1 (1) | 0 (0) | 0 (0) | 0 (0) |
| *ERBB2* | 1 (1) | 0 (0) | 0 (0) | 0 (0) |
| *HRAS* | 1 (1) | 1 (4) | 0 (0) | 0 (0) |
| *CCND1* | 1 (1) | 1 (4) | 0 (0) | 0 (0) |
| *MET* | 1 (1) | 1 (4) | 0 (0) | 0 (0) |

Amplification was established once in KRAS (13 copies), 2 times in EGFR (15 and 103 copies), once in CDK4 (29 copies), once in HRAS (10-20 copies) and once in MET (12 copies).
